# Molecular characterization of *Campylobacter* causing human clinical infection using whole-genome sequencing: Virulence, antimicrobial resistance and phylogeny in Ireland

**DOI:** 10.1371/journal.pone.0219088

**Published:** 2019-07-10

**Authors:** Natalia Redondo, Anne Carroll, Eleanor McNamara

**Affiliations:** 1 Public Health Laboratory, Dublin-Health Service Executive, Dublin, Ireland; 2 European Public Health Microbiology Training Programme (EUPHEM), European Centre for Disease Prevention and Control, Stockholm, Sweden; Cornell University, UNITED STATES

## Abstract

**Objectives:**

We characterized clinical isolates of *Campylobacter* using whole-genome sequencing (WGS) for detection of virulence genes, antimicrobial resistance markers and phylogenetic analysis in order to increase the knowledge on the molecular epidemiology of *Campylobacter* in Ireland, where there are significant gaps due to the widespread in the use of culture independent methods for the diagnosis of campylobacteriosis.

**Methods:**

WGS was applied to 122 *Campylobacter* human isolates collected over a 10-years period, from diarrhoeal stool samples submitted for routine enteric screening.

**Results:**

Genes associated with cytotoxin production such as *cdtA*, *cdtB and cdtC* were found in 88%, 89% and 89% isolates, respectively; adherence, colonization and invasion genes such as *cadF*, *dnaJ*, *racR*, *iam*, *virB11* and *ciaB* were found in 99%, 99%, 98%, 99%, 1% and 80% isolates, respectively. Genetic markers associated with resistance to quinolones (C257T in *gyrA*), beta-lactams (*bla*_oxa-61_) and tetracycline (*tet*(O)) were present in 43%, 71% and 25% isolates, respectively. The *cmeABC* operon was present in 94% of isolates. No macrolide or aminoglycoside resistance markers were detected. Phylogenetic analysis showed that 112 isolates were assigned to 29 sequence types grouped into 17 clonal complexes. Four clusters previously unidentified were detected. These results shown the similarity of Irish data compared to what has been described globally.

**Conclusions:**

WGS has shown a high discriminatory power for cluster detection, demonstrating that its integration in routine laboratory surveillance could improve the detection and management of outbreaks. In addition we were able to demonstrate that virulence genes in clinical *Campylobacter* infections in Ireland were similar to those known previously. High prevalence of quinolone resistance markers has been found, which has implications for antimicrobial stewardship.

## Introduction

*Campylobacter* is a leading cause of bacterial foodborne illnesses worldwide. In 2015, *Campylobacter* was the most commonly reported gastrointestinal bacterial pathogen in human in the European Union (EU) [1]. The species *Campylobacter jejuni* is responsible for 80–90% of cases, while *Campylobacter coli* accounts for 10–20% of human infections. The clinical manifestations caused by *C*. *jejuni* and *C*. *coli* infections are indistinguishable and comprise diarrhoea, abdominal pain and cramps as the most typical symptoms. Infection is mostly acute and self-limited. However, complications such as pancreatitis, cholecystitis, peritonitis and gastointestinal haemorrhage arise in less than 1% of cases and mostly in immunocompromised, the very young or very elderly individuals [2].

*Campylobacter* colonize the gastrointestinal tract of a wide variety of food-producing animals such as poultry, sheep, cattle and swine but also companion animals, wild animals and birds [3, 4]. Campylobacteriosis is a zoonosis, and humans acquire the infection by the consumption or handling of contaminated meat, poultry being the commonest source of infection. Water reservoirs have also been described as possible source of *Campylobacter* transmission [5, 6].

To date, the most commonly used typing methods of *Campylobacter* species are pulsed-field gel electrophoresis (PFGE) and the seven-locus multilocus sequence typing (MLST). However, these methods have limited discriminatory power and provide only typing information. WGS can provide information about subtypes, the presence of virulence genes and antimicrobial resistance markers using a single method, as well as providing higher discriminatory power to identify clusters of related strains which is important in public health laboratories [7].

In Ireland, as in the EU, campylobacteriosis is the commonest cause of gastroenteritis [8]. Currently, due to the widespread use of culture independent methods for the diagnosis of campylobacteriosis in most clinical laboratories since 2015, there are significant gaps in data on the molecular epidemiology of *Campylobacter* infection, with poor or no data on antimicrobial resistance markers circulating in humans isolates. Usually *Campylobacter* gastro enteric infections do not require treatment but in some severe cases treatment may be necessary, with macrolides, such as erythromycin, or quinolone, such as ciprofloxacin, the antibiotics of first and second choice.

The four priority antimicrobial drug resistance of *C*. *jejuni and C*. *coli* by ECDC-EFSA are ciprofloxacin, erythromycin, tetracycline and, since June 2016, gentamicin.[1].

Due to the high diversity of *Campylobacter* sp. the pathogenic mechanisms causing clinical symptoms are still not very well understood. Virulence properties found in *Campylobacter* include motility mediated by flagella, adherence and colonization of the intestinal mucosa, invasion capabilities and cytotoxin production (18).

The aim of this study was to perform a genomic characterization of clinical isolates of *Campylobacter spp*. using WGS in order to provide insights of the presence of 9 virulence genes previously identified as coding for virulence determinants, detection of antimicrobial resistance markers with regard to the priorities in the EU region and phylogenetic relationship of strains collected in the East region of Ireland.

## Materials and methods

### Isolates

A total of 122 human isolates of *C*. *jejuni* and *C*. *coli* isolated from diarrhoeal stool samples submitted to the regional Public Health Laboratory, Dublin-Health Service Executive, Ireland, for routine enteric screening from 2006 to 2016 were included in the study. The isolates had been stored frozen on beads. One bead of each isolate was streaked on blood agar and incubated at 42°C under microaerophilic conditions for 48 hours. Viable isolates were confirmed as *Campylobacter* spp. by real-time PCR using previously described primers targeting the 16SrRNA gene [9].

### Whole-genome sequencing

DNA was extracted on MagNa Pure 96 (Roche Applied Science, Manheim, Germany) according to manufacturer’s instructions. The quantity of the DNA was measured using a Qubit fluorimeter 3.0 (Fisher Scientific, Massachusetts, USA) with the double-stranded DNA (dsDNA) assay HS kit. Libraries were prepared using the Nextera XT DNA kit, according to the manufacturer’s instructions (Illumina, Cambridge, UK). The quality of the library was assessed with a Bioanalyzer 2100 (Agilent Technologies, California, US). Paired-end sequencing was performed on the Illumina MiSeq platform using the v3 reagent kit.

### Data analysis

FASTQ files from sequenced genomes were processed using BioNumerics v.7.6. (bioMérieux, Marcy-l'Étoile, France) calculation engine and the Multi Locus Sequence Type (MLST) client plug-in, which includes core genome MLST (cgMLST) analysis. Assembly-free and assembly-based allele detection analyses were performed for each isolate. The *de novo* assembly was performed using *SPAdes* integrated into the calculation engine. The quality of the assembly was assessed according to different parameters, the average quality score ranged from 31 to 37 (mean, 36), the average read coverage ranged from 31 to 467 (mean, 132), the *N*50 ranged from 6140 bp to 544758 bp (mean, 74417 bp), the number of contigs ranged from 11 to 734 (mean, 116) and the length of the sequences ranged from 1579481bp to 1844823 (mean, 1672005bp). The sequences included in the study were registered with the BioProject database under the BioProject ID PRJNA534408.

### Identification of virulence genes

The detection of the different genes was performed using an *in silico* analysis using the Sequence Extraction plug-in in BioNumerics v.7.6 based on Basic Local Alignment Search Tool (BLAST, National Center for Biotechnology Information, US National Library of Medicine). The minimum sequence identity was set at 80% and the minimum length coverage was set at 90%. The sequences were downloaded in FASTA format from GenBank and then extracted from the *de novo* assemblies of each isolate. In this study the genes *cdtA*, *cdtB* and *cdtC* represented the pathogenic genes encoding for cytotoxin production; *cadF*, *dnaJ* and *racR* represented the pathogenic genes for the expression of adherence and colonization factors, and the genes *virB11*, *iam* and *ciaB* represented the pathogenic genes responsible for the expression of invasion [10, 11].

### Identification of antimicrobial resistance markers

An *in silico* assessment of the presence of antimicrobial resistance markers was performed with the Sequence Extraction plug-in in BioNumerics v.7.6 as described above. For point mutations, ResFinder 3.0 (Center for Genomic Epidemiology) [12] and BLAST were used. The presence of *cmeA*, *cmeB* and *cmeC* as part of the *cmeABC* operon, a multidrug efflux pump conferring resistance to quinolones was studied. The presence of genes *ermB* (erythromycin *resistance)*, *bla*_oxa-61_ (beta-lactam resistance), *tet(O)* (tetracycline resistance) and the cassette *aadE-sat4-aphA3* (aminoglycoside resistance) was investigated. Point mutations such as mutation C257T in the DNA gyrase A gene (*gyrA*), associated with quinolone resistance, and mutation A2075G in the 23S rRNA gene, associated with macrolide resistance, were analysed.

### Phylogenetic analysis

A core genome MLST (cgMLST) spanning tree was created in BioNumerics v.7.6. using categorical differences and the unweighted-pair group method with arithmetic mean (UPGMA). The cgMLST scheme had 1343 loci and 7 MLST for *C*. *coli/jejuni* (pubmlst.org) in BioNumerics v.7.6. and is based on the cgMLST by Cody *et al*. [13] and the 96 publicly available reference sequences of *C*. *coli/ jejuni* [14].

## Results

### Presence of virulence genes

The *cdtABC* operon was present in 105 (86%) isolates and there were no differences between *C*. *jejuni* and *C*. *coli*, with 86% and 89% of isolates presenting the operon, respectively. The genes *cadF* and *dnaJ* were present in 121 (99%) isolates and the gene *racR* was found in 119 (98%) isolates. Genes related to invasion were found as follows: *iam* in 121 (99%) isolates, *virB11* in only 1 (1%) isolate and *ciaB* in 98 (80%) isolates. The prevalence of each gene and its role in pathogenesis are represented in Table 1.

**Table 1:** Prevalence of virulence-associated genes and antimicrobial resistance markers.

Target		Gene	N*	%
Virulence genes	Cytotoxin production	*cdtA*	107	88
		*cdtB*	108	89
		*cdtC*	108	89
	Adherence and colonization	*cadF*	121	99
		*dnaJ*	121	99
		*racR*	119	98
	Invasion	*virB11*	1	1
		*iam*	121	98
		*ciaB*	0	0
Resistance markers	Multidrug efflux pump	*cmeABC*	115	94
	Quinolones	*gyrA* mutation Thr86Ile	52	43
	Macrolides	23S rRNA A2059G	0	0
	Erythromycin	*ermB*	0	0
	β- Lactams	*bla*_*oxa-61*_	87	71
	Tetracycline	*tet(O)*	30	25
	Aminoglycoside	*aadE-sat4-aphA3*	0	0

The presence of virulence-associated genes and antimicrobial resistance markers in *C*. *jejuni* and *C*. *coli* human isolates. PHL-Dublin, Ireland, 2006–2016.

* N = number of isolates.

### Antimicrobial resistance genes and point mutations

The *cmeABC* operon was present in 115 (94%) isolates. The mutation Thr86Ile in *gyrA* was found in 52 (43%) isolates, while no evidence of the mutation A2075G in the 23S rRNA was found. The *ermB* was not detected in any isolates. The *bla*_OXA-61_ was present in 87 (71%) isolates and the *tet(O)* was detected in 30 (25%) isolates. The cassette *aadE-sat4-aphA3* was not detected. The prevalence of resistance markers and its association with antimicrobial resistance are summarized in Table 1.

### Phylogenetic analysis

**Fig 1:**
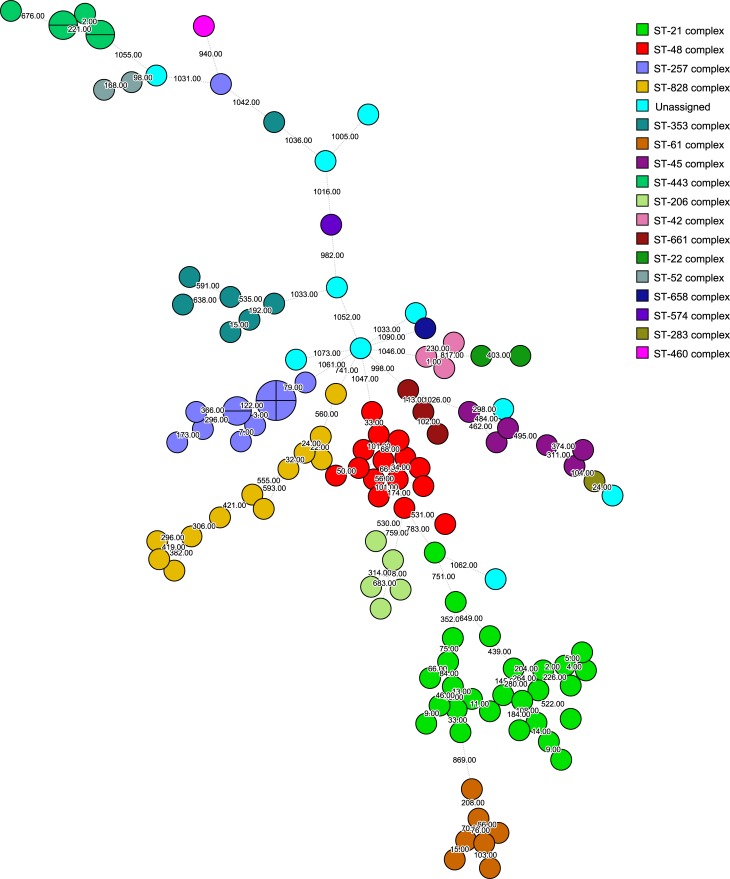
Phylogenetic analysis. Minimum spannin tree (MST) of cgMLST (1343 loci) data of *Campylobacter* human isolates collected from 2006 to 2016 at Public Health Laboratory, Dublin, Ireland. Each colour represents one clonal complex (CC). Isolates are represented by circles. Branches and numbers represent allelic differences between isolates.

**Fig 2:**
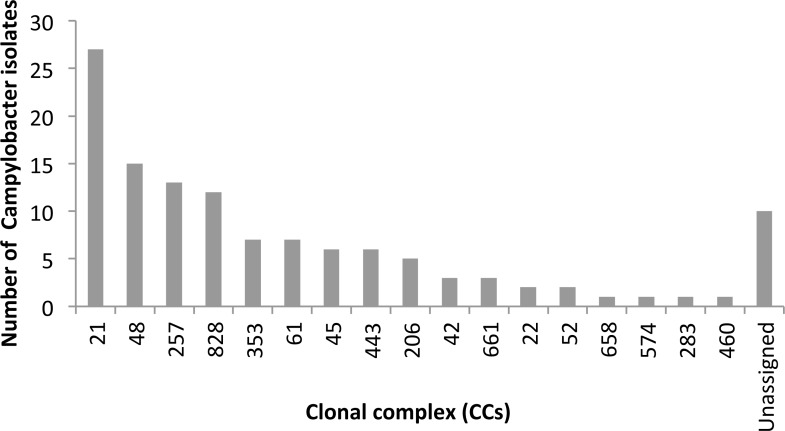
Prevalence of clonal complex. Distribution of CCs amongst 122 clinical *Campylobacter spp*. isolates obtained from 2006 to 2016, PHL-Dublin, Ireland.

Of the 122 study isolates, 112 (92%) were assigned to 29 distinct sequence types (ST) grouped into 17 clonal complex (CC) (Fig 1). Ten isolates (8%) did not belong to a known CC but did include 4 ST (ST2153, ST4831, ST2274 and ST441). The most prevalent CCs (CC21, CC48, CC828, and CC257) accounted for 55% of isolates and the most prevalent STs were ST48, ST21 and ST257, with 14, 11 and 9 isolates, respectively (Fig 2). In addition, four clusters previously unidentified were detected, one with four isolates with no allele differences. There were other three clusters formed by two isolates in each with no allele differences. Further analysis of these clusters showed no obvious epidemiological link among the isolates since they were collected in different years and from different geographical regions.

## Discussion

This is the first genomic study of *Campylobacter* in Ireland using WGS, providing insight into the prevalence of virulence genes, antimicrobial resistance markers as well as relatedness of human clinical isolates.

Pathogenesis of *Campylobacter* is still uncertain, with several virulence-associated genes identified but their role is not completely understood in the development of *Campylobacter* associated gastroenteritis [15]. In our present study, most isolates were positive for the presence of virulence-associated genes related with adherence, colonization and invasion. This was consistent with the findings in previous studies, where genes such as *cadF*, *dnaJ*, *racR*, *iam and ciaB* were also present in high proportion of the isolates studied [10, 16, 17]. The three genes *cdtA*, *cdtB* and *cdtC* are necessary for the functionality of the CDT operon [18] leading to toxin production and cell dystension, however its role in pathogenesis is still unclear. Previous studies in Bangladesh and Turkey have shown high prevalence of CDT in diarrhoeal samples indicating that the toxin production could increase the fluid secretion in the intestine and cause diarrhoea [10, 19]. All the isolates in our study were from diarrheic symptomatic patients, however we did not detect the three genes *cdtA*, *cdtB* and *cdtC* in all isolates (87%, 88% and 88%, respectively), suggesting that other virulence factors could contribute to the clinical presentation of gastroenteric campylobacteriosis, which was also observed in a previous study carried out in Denmark [20].

The gene *virb11*, encoded by the pVir plasmid [21], was found in only one isolate of *C*. *jejuni*. This result correlates with previous findings from Denmark, Bangladesh and Canada, where it was consistently low prevalence [10, 16, 22, 23], suggesting that *virB11* is not an important virulence factor in human infection. Thus, the variability and uncertainty of what virulence factors are associated with clinical *Campylobacter* infection globally was also found in our Irish data. However, monitoring the presence of virulence genes could allow detection of emergent virulence markers (e.g. as a consequence of horizontal transmission), that could lead to more severe campylobacteriosis cases.

Although many limitations exist about reliability of antimicrobial susceptibility testing based on genomic methods compared to phenotypic methods [24], it is recognised that the advantage of knowing what molecular antimicrobial resistance markers are present in bacterial populations is crucial for future development of prevention and control strategies to fight antimicrobial resistance globally. However, such genomic antimicrobial resistance analysis needs validation and standardization prior its applicability in clinical settings as stated recently by the European Committee on Antimicrobial Susceptibility Testing (EUCAST) Subcommittee [24]. In campylobacteriosis, the vast majority of cases have self-limiting symptoms and do not require antimicrobials. However, considering the volume of infections and the increasing acquisition of resistance mechanisms, genomic antimicrobial resistance detection is an essential surveillance tool for better understanding of the resistance markers in *Campylobacter* species circulating in a specific region. Such knowledge will improve the treatment options in the severe cases that do require systemic antimicrobials.

Ciprofloxacin resistance has been linked with the presence of two different markers. One is the presence of the *cmeABC* operon, which is the most common multidrug efflux pump found in *Campylobacter* and contributes to fluoroquinolone resistance by decreasing the amount of the drug in the cells [25, 26]. Other antimicrobial markers are point mutations in the quinolone-resistance determining region (QRDR) of DNA gyrase A (GyrA). In particular, the mutation C257T, which leads to the T86I substitution in GyrA, is the most frequently observed mutation in fluoroquinolone resistant isolates, conferring high-level resistance to these antimicrobials [26, 27]. The high level of fluoroquinolone resistance found in the Irish isolates (43%) is in line with what has been reported by other EU countries [1], and would preclude the use of these antimicrobials for empiric treatment of gastroenteric *Campylobacter* infections. Only if quinolone susceptibility is confirmed are such antimicrobials recommended for therapy if indicated.

Regarding macrolides, the most common class of antibiotics for *Campylobacter* infections treatment, two antimicrobial markers have been linked to resistance, the point mutation A2075G in the 23S rRNA gene and the *ermB* gene [6, 26, 27]. The absence of macrolide resistance in isolates of this study would make these antimicrobials the preferred Irish empiric treatment of choice for severe campylobacteriosis. However it is important to take into account that macrolide resistance has been mostly detected in *C*. *coli* isolates as confirmed by EFSA [1]. Since we only had 9 *C*. *coli* isolates in this study this macrolide data should be interpreted with caution.

Tetracycline resistance in *Campylobacter* is conferred by the gene *tet(O)* [26, 28] and it is of concern due to the widespread use of this antimicrobial in the poultry industry globally [29, 30]. The average percentage of *Campylobacter* tetracycline resistance in the EU is 68.8% in human isolates of *C*.*coli* and 44.6% of *C*. *jejuni* [1]. The Irish clinical isolates present lower levels (25%) of tetracycline resistance but continuous monitoring of this antimicrobial resistance genetic marker could aid in alerting the need for ‘One Health’ rapid implementation of reduction and control strategies in tetracycline usage, if an increase in resistance were observed in the future.

Since 2016, gentamicin resistance is included in the EU surveillance objectives to monitor invasive *Campylobacter* disease and trends in resistance in clinical human isolates [1]. In this study we analysed the presence of the gene cluster *aadE-sat4-aphA3*, which has been described in *Campylobacter* isolates from chicken and broilers in China and it is associated with resistance to multiple aminoglycosides. They have suggested that *Campylobacter* might have acquired the gene cluster from a Gram-positive organism [31]. Globally the resistance levels to gentamicin are still very low at EU level, with 0.8% and 1.6% *C*. *jejuni* and *C*. *coli*, respectively [1], correlating with the absence of such resistance in our Irish isolates. However, the emergence of aminoglycosides resistance in both US and China [31, 32] would require the continuous monitoring of such antimicrobial resistance, consistent with the EU strategy. The detection of 71% of Irish isolates with *bla*_oxa-61_ genes linked with beta-lactam resistance was not unexpected considering the usually high prevalence in *Campylobacter* isolates globally. However, this is not of significance considering that β-lactam antibiotics have limited application for treatment of *Campylobacter* infections.

In this study we have also highlighted the wide diversity of *Campylobacter* strains circulating in Ireland. The distribution of CCs among the sequenced isolates was very similar to what has been found in UK, with the CC-21 the most predominant CC, including more than 20% of the isolates, followed by CC-48, CC-257 and CC-828 [33]. These four CCs accounted for more than 50% of the isolates. The predominance of CC-21 has been also observed in other countries such as Israel [34]. In addition, comparing our human results with data from an earlier study with poultry isolates of *Campylobacter* collected in Ireland, there is an overlapping of the three most prevalent ST types [35] in both studies, suggesting a potential active transmission of *Campylobacter* through the food chain over time.

The isolates included in this study were not part of any notified outbreak, however the high discriminatory power of the WGS has brought to light the presence of 4 previously undetected clusters. Thus confirming the advantageous role of genomic characterisation of clinical *Campylobacter* isolates could play in aiding public health identification and investigation of linked cases.

This study has some limitations; it is not based on sentinel or continuous surveillance of clinical campylobacteriosis, but rather on a regional clinical laboratory database of stored isolates, which may not be representative nationally.

This study was confined to human isolates and does not include animal or food isolates, thus the postulated role of the food chain in transmission can only be hypothetical. However further ‘One Health’ *Campylobacter* studies are achievable and will help to inform future public health strategies for the reduction of campylobacteriosis. In addition, the data presented could not differentiate between *C*. *jejuni* and *C*. *coli* due to the unequal predominance of *C*. *jejuni* isolates compared to the low number of *C*. *coli* isolates (less than 10%) in the study dataset. Thus species-specific analysis particularly related to antimicrobial resistance could mislead and potentially leads to an underestimation of the antimicrobial resistance markers. Genotypic compared to historic phenotypic antimicrobial resistance data was not feasible because it was not comprehensive and not contemporaneous.

In conclusion, this WGS *Campylobacter* study has provided very useful additional information on the characteristics of *Campylobacter* isolates from human samples in Ireland. Thus confirming the presence of virulence genes in *Campylobacter* infections in humans. In addition, valuable information on genomic antimicrobial markers circulating in Ireland has been provided, which could contribute in the future to EU data. Finally, our study has shown the presence of clusters previously unidentified and the high diversity of *Campylobacter* circulating in the country, which along with future routine surveillance, could support the implementation of more effective One Health intervention strategies focused on the prevention and control of cases of campylobacteriosis.
